# On a Special Acid of the Lungs

**Published:** 1852-09

**Authors:** 


Jour. de Chemie Medicale, 1852, No. 3.

*On a Special Acid of the Lungs.* By MM. DUMAS & VERDEIL.

M. DUMAS recently presented a paper to the Academy, giving an account of his and M. Verdeil's researches on a special acid secreted by the pulmonary parenchyma in most animals; and which may be found free, but is usually combined with a salt of soda. Obtained in the crystalline form, it is a brilliant body, strongly refracting light. It does not lose its water of crystallization at a temperature of 100° Cent.; and when heated still more, it decrepitates, melts, and is decomposed, giving rise to empyreumatic products. Much coal remains, which disappears without leaving any traces of ash. It is soluble in water and boiling alcohol; but not in cold alcohol or ether. Its ultimate analysis exhibits definite proportions of carbon, hydrogen, oxygen, nitrogen, and sulphur. It forms crystallized salts with bases, and expels carbonic acid from the carbonates.

The existence of this substance is of high physiological interest; for the acid thus secreted by the parenchyma comes in contact with the carbonate of soda of the blood transported by the capillaries, and decomposes it, uniting with the soda and setting free the carbonic acid which is exhaled. The presence of a portion of this acid in the free state, in the parenchyma, indicates that it is really here that it is formed, and not in the blood, which is an alkaline fluid. By uniting with the soda of the blood, the acid does not change the re-action of that fluid, since it merely takes the place of the carbonic acid which is expelled during expiration.

